# Quantitative Computerized Assessment of the Degree of Acetabular Bone Deficiency: Total radial Acetabular Bone Loss (TrABL)

**DOI:** 10.4061/2011/494382

**Published:** 2011-10-17

**Authors:** Frederik Gelaude, Tim Clijmans, Hendrik Delport

**Affiliations:** ^1^Mobelife R&D, Kapeldreef 60, 3001 Leuven, Belgium; ^2^Department of Orthopaedics, AZ Nikolaas, Lodewijk De Meesterstraat 5, 9100 Sint-Niklaas, Belgium

## Abstract

A novel quantitative, computerized, and, therefore, highly objective method is presented to assess the degree of total radical acetabular bone loss. The method, which is abbreviated to “TrABL”, makes use of advanced 3D CT-based image processing and effective 3D anatomical reconstruction methodology. The output data consist of a ratio and a graph, which can both be used for direct comparison between specimens. A first dataset of twelve highly deficient hemipelves, mainly Paprosky types IIIB, is used as illustration. Although generalization of the findings will require further investigation on a larger population, it can be assumed that the presented method has the potential to facilitate the preoperative use of existing classifications and related decision schemes for treatment selection in complex revision cases.

## 1. Introduction

**Classification Systems**

Numerous classification systems have been applied to describe bone deficiencies associated with failed acetabular prostheses, each differing somewhat in purpose and detail. The aim of using a classification system is to predict the nature of the bone deficiency in advance of surgery to allow adequate treatment selection and reconstruction planning. Another important role of these classifications is the promotion of uniform surgical results measurement and reporting [1, 2].

Commonly used acetabular classification systems are for example those developed by Letournel [3], Paprosky et al. [4], and D'Antonio et al. [5]. Throughout the years, the validity and reliability of classification systems have been studied, and altered—possibly improved—systems have been introduced [6–8].

The class definitions are generally spoken qualitative in nature. No direct quantitative input or output measure is used, and, therefore, intraobserver and interobserver reliability is low. Some studies even suggest that, in particular for the acetabulum, bone stock loss classification systems are simply inconsistent and unreliable [6, 7, 9, 10]. Classifications mainly rely on preoperative X-ray images, complemented with intraoperative findings. In case of doubt, also preoperative computed tomography (CT) scan information can be involved, either by direct inspection of the planar slice images or from an overall reconstructed three-dimensional (3D) visualisation which has been directly generated in the CT scanner software.

Current use of the aforementioned imaging modalities can be problematic. X-rays for example merely present a scaled projection of the three-dimensional reality; while the aforementioned 3D CT visualisation mainly highlight the failed metallic implant components and do not provide a clear view of the remaining bony situation.

In contrast, classification systems are based upon and even illustrated by 3D images of hemi-pelves, in total absence of failed components. Up till today, while using X-rays and direct 3D scanner visualisations, the only occasion in which the classification could truly be applied occurs during surgery, after making the incision and effectively removing the failed components. This leaves little time to set the actual diagnosis. And; furthermore, the choice of implant solution is inherently restricted to readily available off-the-shelf standard implants, as really custom-made implant solutions are implicitly excluded.

After all, really customized implant solutions, such as a three-flanged implant for acetabular reconstruction [11, 12], possibly combined with structural and/or morselised allografts and possibly used in combination with bone quality analysis and screw guidance jigs, requires a precise 3D-image-based preoperative planning, and, thus, entail a lead time—ranging from just a couple of weeks to numerous months depending on the supplier.

In response, modular systems with standard defect filling augmentation components presently gain popularity [13]. However, especially for the extremely wide defects such as Paprosky's types III, the question remains if the optimal solution is selected. This in view of long-term stability and restoration of functionality whereas the assembled construct, which consists out of multiple separate components, should fill and span the wide and often uncontained defect and engage closely to the specific surrounding bony situation, and also in consideration of preserving the few remaining but extremely valuable bone stock, whereas modular systems are standard in shape and thus may require reaming.

A precise preoperative classification of bone loss would enable the clinician to make a proper selection from a wide and unlimited range of implant solutions.

This paper presents a novel *quantitative*, *computerized, *and therefore highly objective method to assess the degree of bone deficiency in the acetabulum. The starting point is the 3D bone model of the bony anatomy without any metal, meticulously segmented from the CT scan data.

Goal of the novel method is to facilitate the preoperative use of existing classifications and any directly related decision schemes which are used for clinical treatment selection. An example is the scheme described by Paprosky et al. [14], which focuses on the ability of remaining host bone to provide initial stability to a hemispherical cementless acetabular component. In fact, the newly proposed method will quantitatively characterize bone presence around a comparable (hemi)sphere. 

The proposed method makes uses of advanced 3D CT-based image processing and effective 3D anatomical reconstruction methodology. Both methodologies used in this paper, including the respective validations, have been presented previously in the literature [15, 16]. Nevertheless, it should be stated that similar validated methods from other research groups can be applied [17–24]. 

The output of the quantitative bone loss assessment tool will consist of a ratio, as well as a graphical representation. Both will allow direct comparison between deficient and/or healthy hemipelvis cases.

## 2. Materials and Methods

**Figure 1:**
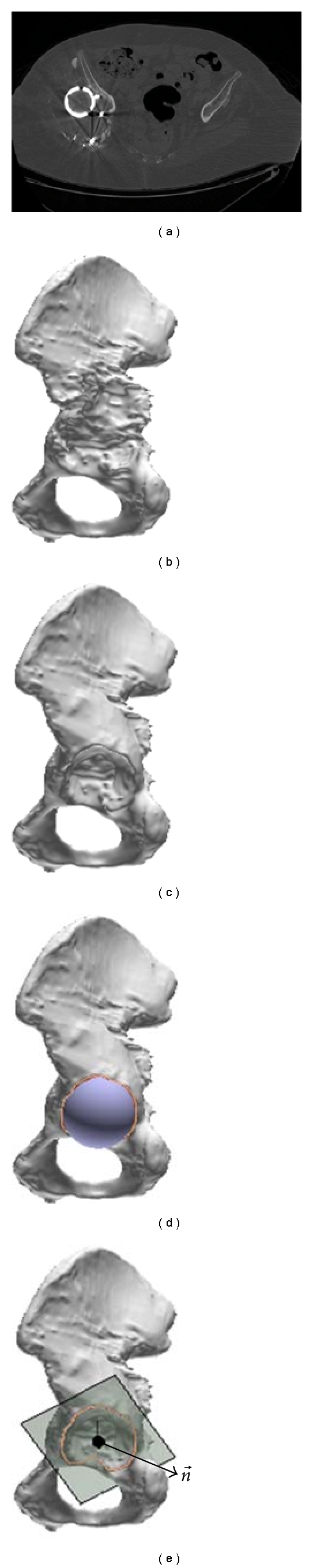
Image processing, bone model extraction and reconstruction. (a) CT dataset (only one slice image shown). (b) 3D bone surface model. (c) Reconstructed 3D bone surface model. (d) Fit sphere on outlined acetabular region. (e) Fit plane on acetabular rim, with acetabular axis through sphere center point in plane normal (outgoing) direction $\vec{n}$.

First, a CT scan is acquired and processed in a dedicated image processing software (Mimics, Materialise NV, Leuven, Belgium) (Figure 1(a)). A three-dimensional bone surface model of the defective hemipelvis is then calculated (Figure 1(b)). (STL mesh, Standard Triangulation Language, Marching Cubes algorithm [17], accuracy settings in accordance to Gelaude et al. [15]). 

Secondly, given the three-dimensional shape of the defective acetabulum of the patient, a computer algorithm is applied to obtain the anatomically reconstructed shape of the acetabulum. The algorithm used in this paper first applies a set of mirroring and matching operations with respect to a normal contralateral or database bone and then integrates the missing bone geometry into the original bone mesh [16]. Alternatively, a statistical shape algorithm can be applied [23] based on a database of hemi-pelves [18–22, 24]. 

The result is a three-dimensional surface model of the anatomically reconstructed bone (Figure 1(c)). A single user interaction takes place by outlining the rim of the reconstructed (healthy) acetabular region (Figure 1(d)).

The following steps are processed to obtain a distance deviation map and related ratio for the degree of total radial acetabular bone loss (TrABL) in the acetabulum. The actual implementation of the method is performed in the Matlab programming environment (The Math Works Inc., Natick, Mass, USA).

### 2.1. Assessment of Total radial Acetabular Bone Loss

**Figure 2:**
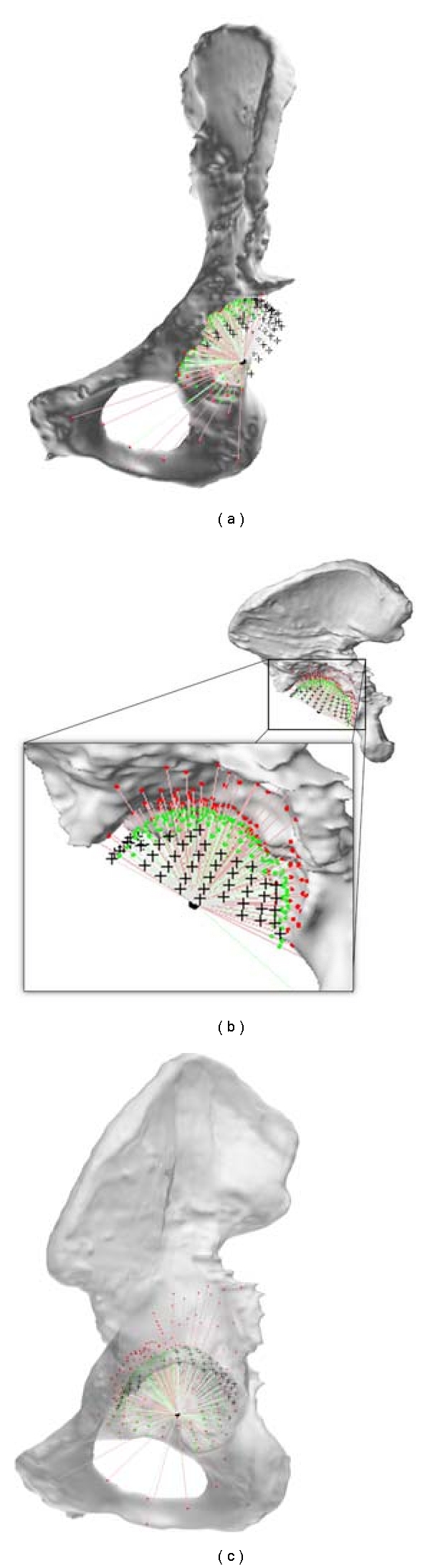
Ray entry points. Red: on the deficient bone mesh (a-b). Green: on the reconstructed bone mesh (c). Black points result from rays that intersect only with reconstructed bone mesh, that is, total bone loss in direction of those rays (Note: bone model in (c) is displayed transparently; (b) is displayed with a zoom box.)

On the reconstructed 3D bone surface mesh, the joint center can be located by fitting a sphere in the reconstructed healthy acetabulum [16] (Figure 1(d)). From this point, a set of uniformly distributed rays is defined in radial outward direction, similar to an explosion of rays from a central point, in this case being the center of the reconstructed acetabulum. The rays can intersect the original deficient bone mesh as well as the reconstructed bone mesh (Figure 2).

In particular in regions with bone deficiency, the rays first penetrate the reconstructed (intact) mesh and, only further on intersect the deficient mesh. Or, in some regions, the rays only intersect the reconstructed mesh and simply pass by the deficient mesh. 

The distance deviation between the two entry points of a ray reflects the severity of bone loss in direction of the ray. The Euclidian distance can vary from very small (zero in case of coinciding meshes, i.e., no deficiency) up to very large and even up to infinity if no intersection with the deficient mesh was detected. The distance measure reflects the availability of bone stock in radial direction, as defined by the rays. If the distance measure is infinite, the defect is simply not contained in that direction.

**Figure 3:**
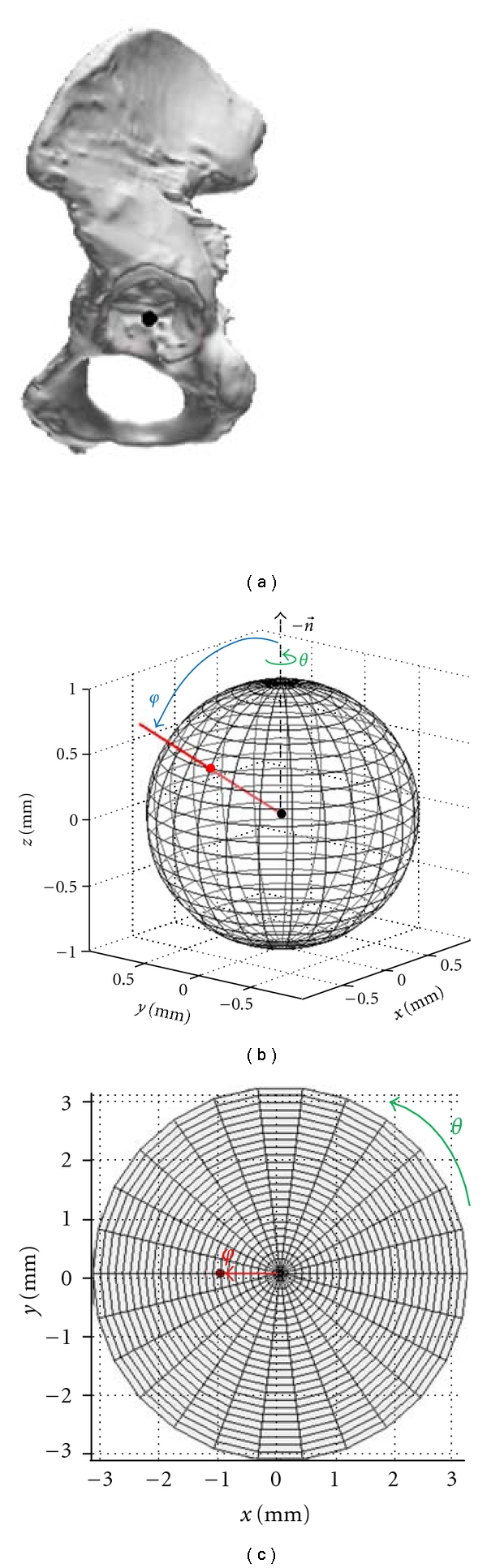
Definition of unit sphere and projection of the unit sphere into a specific planar grid. (a) Reconstructed mesh with unit sphere located at the center of the acetabulum (see Figure 1(d)). (b) Initial unit sphere in three dimensions, with ray (red line) from center point (black) to grid point (red). (c) Planar grid representation, with the example grid point highlighted (red) ($\vec{n}$: acetabular axis, see Figure 1(e), *θ*  and  *φ* according to formulas (1) to (2).)

The 3D distance deviations can be displayed in a simple and clear manner, on a grid. The grid is derived from a unit sphere (sphere with radius 1 mm) located at the center of the reconstructed acetabulum (Figures 1(e) and 3(a)):

(1) $$\begin{array}{cc} x & =r_{u}*\sin\;\;\left(\theta \right)*\cos\;\;\left(\varphi \right),\\ y & =r_{u}*\sin\;\;\left(\theta \right)*\sin\;\;\left(\varphi \right),\\ z & =r_{u}*\cos\;\;\left(\theta \right), \end{array}$$

with spherical coordinates *φ* ∈ [0, *π*], *θ* ∈ [−*π*, *π*], and *r*_*u*_ = 1 mm. Note that rays with *φ* = 0 are oriented along the acetabular axis into the middle of the reconstructed acetabulum. This corresponds to vector $-\vec{n}$ (see Figure 1(e)).

Rays are mathematically defined by the grid points and, collectively, point away from the single center point. The grid on the sphere is then converted (unfolded) into a planar grid, as can be obtained by azimuthal projection—a method routinely used in cartography. In particular, a polar to Cartesian coordinate transformation is performed, as follows:

(2) $$\begin{array}{cc} x & =r*\cos\;\;\left(\theta \right),\\ y & =r*\sin\;\;\left(\theta \right), \end{array}$$

with  *r* = *r*_*u*_∗*φ*, following the definition of arc length.

Units for *φ* and *θ* are defined in radians, for *x*, *y*, *r*_*u*_, and *r* in millimeters.

**Figure 4:**
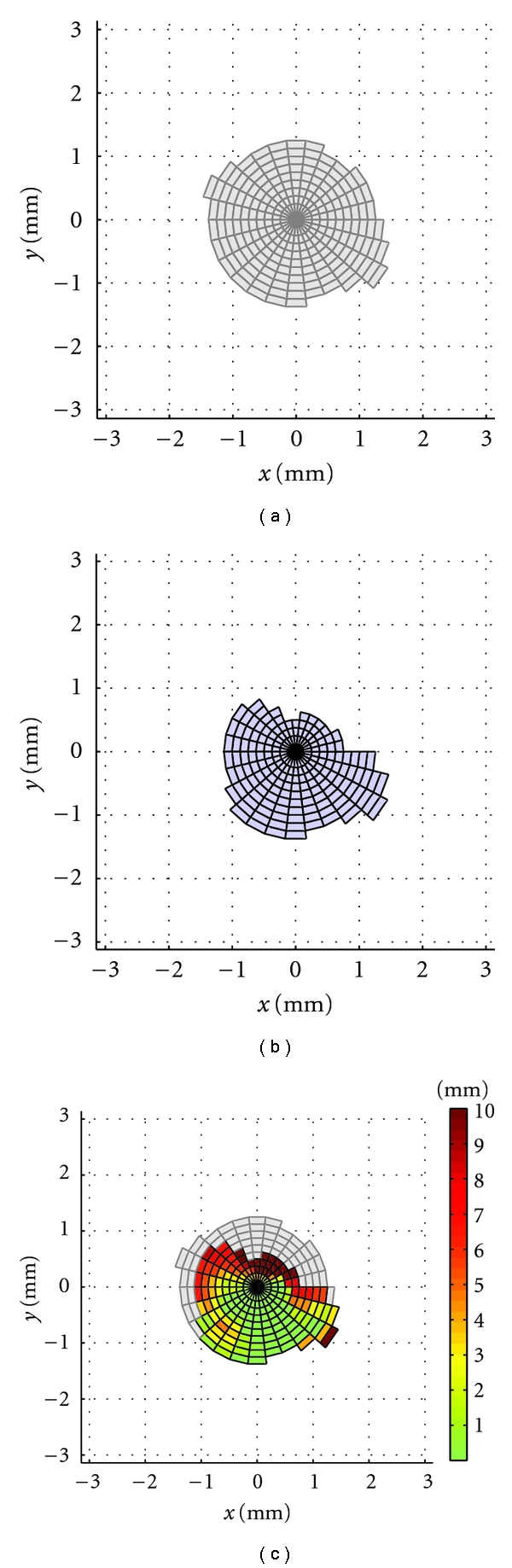
Total radial acetabular bone loss (TrABL) graphs. (a) and (b) Obtained by intersecting the reconstructed and deficient (patient) bone mesh, respectively. The grid being obtained from a unit sphere located at the center of the reconstructed acetabulum folded open into a planar grid (formulas (1) to (2)). The proportion of the surface area of both colored surfaces (uniform grey and blue) is denoted the TrABL *ratio *(see formula (3)). (c) Overlay view of (a) and (b), with a color range for the calculated Euclidean distance deviations applied to (b). This is the TrABL *color graph.* For this paper, a color range from 0 mm (green) to 10 mm or higher (dark red) is adopted. (Note: a moderate sampling rate of 25 × 25 sphere points is applied to generate these figures, to enable clear interpretation.)

On the grid, two surfaces are plotted: one surface enclosing all grid points that correspond to rays that actually intersect the reconstructed bone mesh and one surface, in turn, for the deficient bone mesh. The former is displayed in grey color (Figure 4(a)), while the latter is assigned a blue color in Figure 4(b). Both graphs can be combined; and, based on the deviation values and on a predefined color range, a color graph can be shown on the surface relating to the deficient bone mesh. This is illustrated in Figure 4(c). 

The degree of total bone loss in the deficient bone mesh can be expressed in a quantitative way by comparing the surface areas of both enclosed surfaces on the planar grid. The ratio of the grid surface area of the deficient mesh intersections A_def_ to the grid surface area of the reconstructed mesh intersections A_rec_ is designated the Total radial Acetabular Bone Loss (TrABL) *ratio*. This ratio is a measure for the amount of original acetabular bone that is completely missing in the deficient acetabulum in comparison to the reconstructed acetabulum,

(3) $$\begin{array}{c} \text{TrABL}\;\text{ratio}=\left(1-\frac{A_{\mathrm{def}}}{A_{\text{rec}}}\right)*100\%. \end{array}$$

The *TrABL color graph*, which is displayed on the projected grid, indicates whether or not bony support in radial direction can be found. If not, the color is grey. If yes, a distance is known, and the color is taken from a color range (Figure 4(c)).

Mutual comparison of distance maps between different patients or for the same patient on different occasions is possible on two conditions: the use of a fixed grid and a fixed color range for the deviations. The first condition is met by default using the unit sphere and applying a fixed sampling rate for the sphere/grid of 100 × 100. The second condition is user defined. In this paper, a color range from 0 mm (green) to 10 mm or higher (dark red) will be adopted for the reported retrospective clinical examples.

### 2.2. Definition of Anatomical Subregions

The TrABL ratio can also be assessed in predefined subregions of the grid; each region will correspond to a particular anatomical region oriented around the joint. Since—to our knowledge—no standard and/or computerized subdivision of the acetabular region in three-dimensional space has been described in literature—DeLee and Charnley [25] and Gruen et al. [26] did it for X-ray for acetabulum and femur, respectively—the following 3D approach was defined. 

On the reconstructed bone surface mesh, the joint center can be located by fitting a sphere in the reconstructed healthy acetabulum. This region was already outlined by the user in a single interaction moment (see above).

A plane is fit onto the acetabular rim (least squares algorithm) (Figure 1(e)). The acetabular axis $\vec{n}$ is defined by the sphere center point and the plane normal and points into lateral (outgoing) direction.

**Figure 5:**
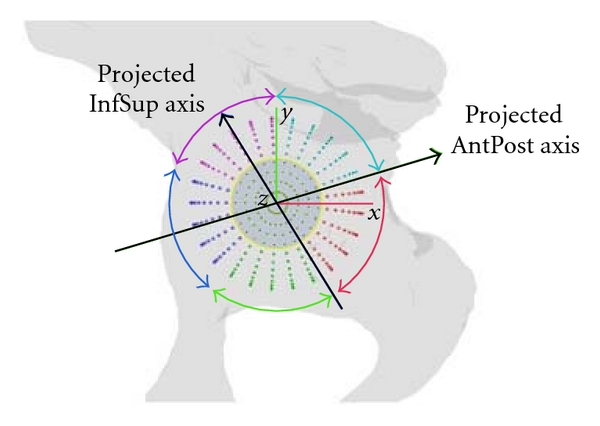
Subdivision of acetabular region. All data, including the anatomical axes, are projected in the acetabular rim plane (see Figure 1(e)). Then a subdivision for the sphere points is made around the center point (medial region) and for five regions around the projected anteroposterior (AntPost) anatomical axis. (Note: for visualisation purposes, shown for enlarged sphere, not unit sphere.)

One region encompasses the medial points, being all unit sphere grid points lying within a distance that equals half the radius of the unit sphere, from the acetabular axis. The remaining grid points are then subdivided into five regions spread equally around the anteroposterior anatomical axis of the patient, all data being projected in the acetabular plane (Figure 5). The anatomical axis is derived from pelvic anatomical landmarks in accordance to the ISB standardization [27]. Computerized landmark detection (ASIS line and pubis point in particular) is adopted as described by Gelaude [28]. Eventually, the following six regions are obtained: posteroinferior (PostInf), inferior (Inf), anteroinferior (AntInf), anterosuperior (AntSup), posterosuperior (PostSup), and medial (Med).

### 2.3. Data Collection: Illustration of the Method

Twelve CT scans of patients in need for revision of the hip joint were selected retrospectively and at random (6 male, 6 female; mean age 65.3 ± 9.0 yrs) (CT slice increment ranging from 1.25 to 3 mm, in-plane voxel size from 0.6 to 0.9 mm) (time period of selection from March 2009 till January 2011). 

Eleven patients were diagnosed by the surgeon (HD) as type IIIB [4, 14]. 

For illustration purposes, patients with deficiencies in varying anatomical regions were selected. After all, the presented bone loss quantification tool should be able to characterize each bone loss pattern. With the same purpose, one additional (twelfth) patient with just a type IIc defect—due to protrusion in osteoporotic bone—was included. 

First, the scan data were processed, resulting in 3D bone models of the deficient hemi-pelves specimens. Subsequently, anatomical reconstruction was applied, bringing about the corresponding 3D reconstructed bone models. Finally, the TrABL ratio and graph were calculated for each of the specimens. 

## 3. Results and Discussion

The 3D bone models of the deficient hemi-pelves are displayed in Figure 8 (in red). The corresponding 3D reconstructed bone models are shown in overlay in the same figure (in green, transparently). 

**Figure 6:**
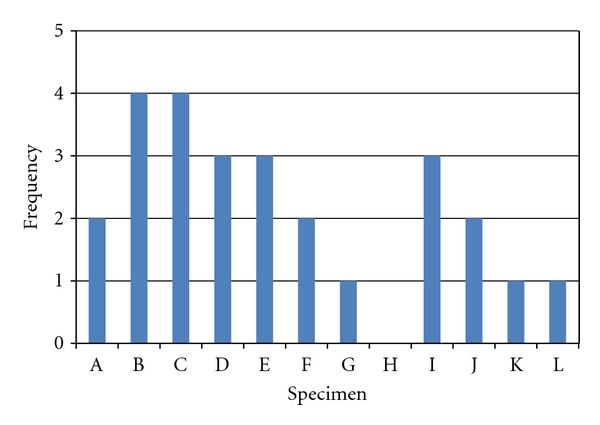
Histogram of TrABL ratios exceeding 25%, per specimen. (Twelve specimens named A to L, as specified in Table 1.)

**Figure 7:**
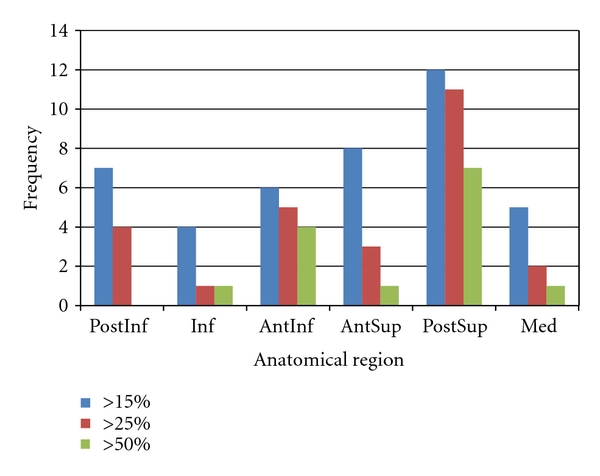
Histogram of TrABL ratios exceeding 15, 25, and 50%, per anatomical region (PostInf: posteroinferior, Inf: inferior, AntInf: anteroinferior, AntSup: anterosuperior, PostSup: posterosuperior, Med: medial).

**Figure 8:**
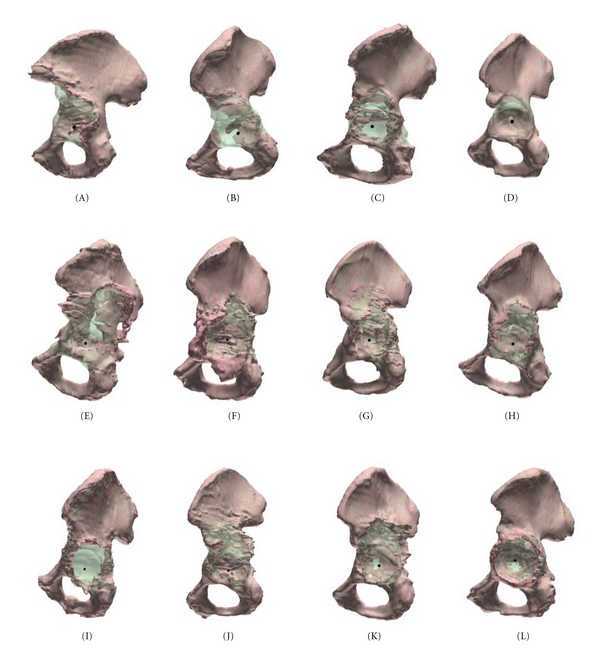
Three-dimensional bone meshes of hemipelvis (twelve specimens named A to L, as specified in Table 1). Red: deficient situation. Green: anatomical reconstruction proposal (transparent and in overlay).

The TrABL ratios are listed in Table 1. These numbers show that all specimens have lost about a quarter of the ideally available bone stock for at least one anatomical region. For five specimens, even, the same minimal degree of total bone loss presents in three regions or more (Figure 6). The histogram in Figure 7 shows that, for the given set of specimens, total bone loss is predominant in the PostSup region. Severe and moderate total bone loss (>50% and >25%, resp.) is the second most frequent in the AntInf region. If the threshold is put at 15% (slight bone loss), the AntSup region takes the second place. 

**Figure 9:**
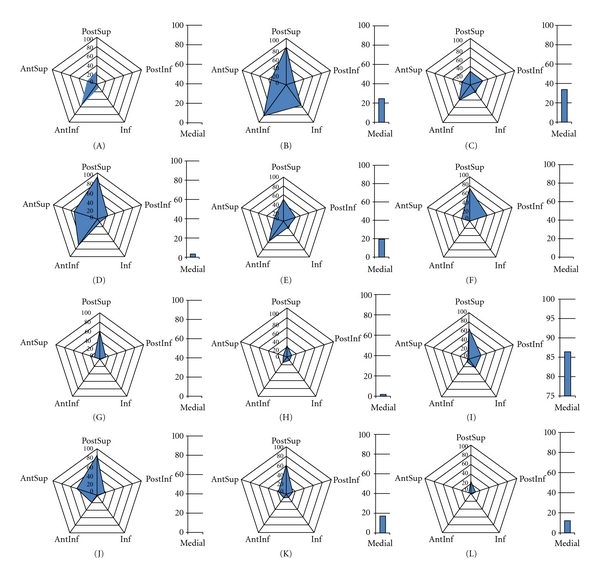
Radar plots of TrABL ratios per anatomical subregion (for twelve specimens A to L, as specified in Table 1) (PostInf: posteroinferior, Inf: inferior, AntInf: anteroinferior AntSup: anterosuperior, PostSup: posterosuperior.) The ratio for medial subregion (Med) is added with a separate bar graph.

In Figure 9, the tabulated ratios per region are graphically presented in a radar plot, for each of the specimens. (Note: this graphical method of displaying multivariate data is independent of the above-presented TrABL color graph of Figure 4(c).) These plots clearly show that total bone loss is mostly multidirectional but with presence of one or two predominant directions. Furthermore, if two predominant directions are present, these often point in opposite directions. For example, for specimens B, D, and E, the AntInf direction is the antagonist of the PostSup direction. 

**Figure 10:**
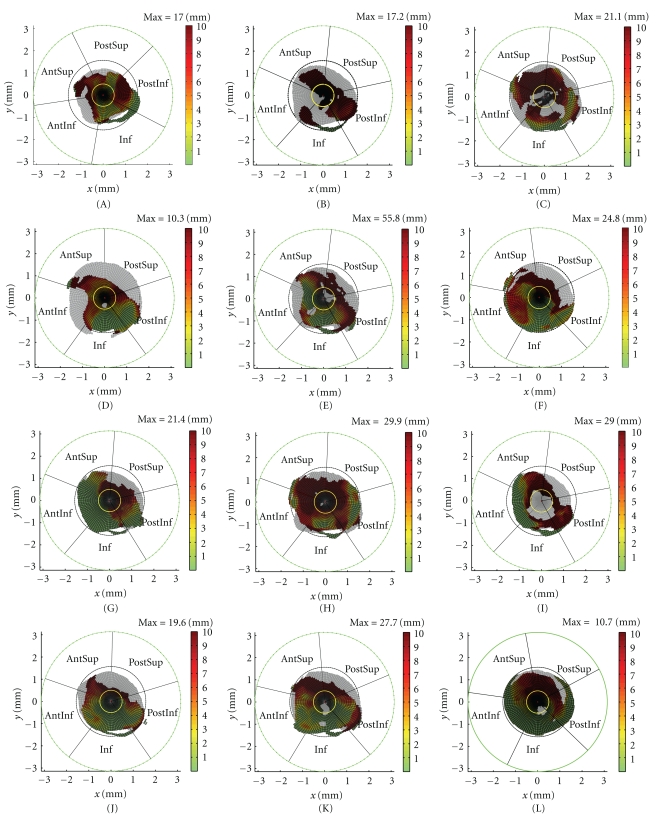
TrABL color graphs (twelve specimens A to L, as specified in Table 1 and as defined in Figure 4) (Yellow circle delineates medial region. Black and green circles represent *φ* = *π*/2 and *φ* = *π*, respectively (cfr.  Figure 3).) The black radial lines outline the anatomical subregions for each specimen (PostInf: posteroinferior, Inf: inferior, AntInf: anteroinferior, AntSup: anterosuperior, PostSup: posterosuperior, Med: medial).

Figure 10 presents the TrABL color graphs. Each specimen appears to have a characteristic bone loss pattern. This can be described by looking (i) at the outline of the regions, that is, in respect to total bone loss and thereby ratio as well as by (ii) inspecting the distance deviations from the ideal anatomical situation when bone is still present (i.e., the coloring of the surface). This will be illustrated on the dataset of twelve specimens. It should be noted that generalizations of the subsequent findings will be the topic of further research, by analyzing a larger dataset.

**(i) The TRABL Surface Outline**

(1) In *healthy specimens* (i.e., looking at the grey surface in the TrABL graph), the perimeter is convexly curved and undistorted and lies within and closely to the circle *φ* = *π*/2 in the TrABL graph (see Figure 10). No holes in the surface are present, with one exception: inferiorly, a small strip may detach from and re-insert in the main surface, thereby forming a “needle head” feature. As illustrated in the lower part of Figure 2(a), this detachment of the surface is caused during ray calculation by presence of the obturator foramen and by the horseshoe shape of the acetabulum. Rays can slip out of the acetabulum and insert (and thereby detect bone) on the lower pubic ramus.

(2) In *deficient acetabuli,* the TrABL surface is distorted by holes, is reshaped or deformed, and may become divided into partitions. For the twelve specimens observed in this study, mostly a combination of all four mechanism occurs.

Holes originate mostly from medial bone loss. At first, possibly on multiple but smaller locations (e.g., specimens D, H, K, L), and eventually leading to a large opening (specimen I).

Reshaping can be observed in specimens B and D, where bone is degraded (planed away) over a large region at once. A large portion of the acetabular rim is missing, but a smooth transition to the surrounding bone stock remains.

Deformation originates from forceful direct or indirect action of failed implant components or by surrounding bones such as proximal femur. The rim and surrounding bone are directly torn open and pushed away, leaving a highly irregular surface on the bone as well as on (the contours of) the TrABL graphs (e.g., specimens E, F, G, H, K, and L). In specimen L, where degradation of the medial wall was just initiated, the cup protrudes the acetabulum, pushing the acetabular rim to the side but also backward in opposite (lateral) direction. The rim here is pushed to above the level of the ideally reconstructed acetabulum. Correspondingly, the TrABL surface grows slightly and possibly goes outside of the circle *φ* = *π*/2 in the TrABL graph.

(3) Partitioning reflects fractures and (more likely) bone degradation resulting in discontinuity of the pelvic bone. In specimen B; for example, the AntInf and Inf portions of the surface are only connected to the other part of the pelvis through the lower pubic ramus (the “needle head”, see above), and at the border between the PostSup and PostInf regions; the connection in the surface is deteriorated; small holes are present at that location. Another example is given by specimen C, where the superior and inferior pelvic parts are merely connected by a small surface transition from PostSup to PostInf, and little time remains until complete dissociation will occur. The diagnosis of pelvic dissociation for specimens B and C was confirmed intraoperatively.

**(ii) The TRABL Surface Color**

The colors in the TrABL surface display important information that is complementary the TrABL surface outline and/or unity of the surface. This can be illustrated by specimens H and L. The TrABL ratios are—to some extent—highly similar, resulting in very similar radar plots in Figure 9. But the dark red colors in the TrABL color graph of specimen H indicate larger distance deviations from the ideal (what would have been the original) reconstructed situation. So the joint cavity of specimen H is eroded to a much larger depth and, therefore, much weaker. Furthermore, the dark colors are spread around the full hemisphere, not limited to the AntSup, PostSup, and PostInf regions.

Another illustration comes from the TrABL surface color of specimen B, which shows that the surrounding bone is very distant compared to other specimens, for example, C.

The presented tool and study has some limitations. *Firstly*, although the number of user interactions is limited, the method is not fully computerized. Namely one interaction is needed in which the user outlines the rim of the reconstructed acetabulum. With this, variation can be introduced in the acetabular direction (and thereby definition of the anatomical subregions) and in the location of the center point of the analysis. A first preliminary analysis of the inter- and intrauser variability suggested a deviation of about 3 percent to one of the TrABL ratios in one of the anatomical subregions. A full in-depth assessment of the variability is scheduled for future research.

A difficulty, however, remains the collection of a representative set of specimens. The same accounts—and this is the *second* limitation—for the generalization of the findings formulated based on the presented dataset of twelve specimens. Collaboration with other research groups is, therefore, indicated. This can be done on a prospective as well as on a retrospective basis.

*Thirdly*, the assessment method is limited to hemipelvis bones with intact ASIS line, which implies inclusion of the contralateral side in the CT scan and with intact pubis. For IIIB defects, the aforementioned anatomical regions are mostly intact, and, in specialized centers, a CT scan is mostly taken by default to prepare for surgery. The solution will be to introduce and pursue a clear but simple scan protocol. For larger defect sizes, such as in oncology, the need for assessment of bone loss is very limited or inexistent. That type of surgery involves resections and is not by definition bone sparing. For smaller defect sizes, CT will justly not be available due to irradiation protection of the patient. Future research can look into deriving an instant assessment tool for simple revision cases, possibly 3D but still X-ray based [29].

*Fourthly*, the presented tool is not instant. Or, to be more exact, it runs smoothly—1 minute on a standard computer—but requires time for preparatory steps such as image segmentation and defect reconstruction. Typically this takes about one working day and may depend on complexity of the case. Fortunately, this preparatory work gradually becomes standard practice for any adequate planning and/or related guiding technique such as personalized drill jigs and image-guided navigation for complex cases. Also, this work can be outsourced to technicians, so the surgeon can keep his focus on decision making and patient treatment. Furthermore, social reimbursement instances and/or private insurance decision makers in dossiers of novel technology for treatment of complex (costly) revisions are very in favor of objective classification schemes and clear documentation about the 3D bony situation of the patient. And finally it is the best interest of the technology, the surgeons, and the patients to document the population and cases by applying classifications systems that are as refined and objective as possible.

*Fifthly*, the accuracies of the preparatory steps—image segmentation and defect reconstruction—are determinant for the accuracy of the TrABL ratio and graph. Looking into *image segmentation accuracy*, the literature shows that 3D bone surface meshes can be generated with mean absolute accuracies of up to one-fifth of the voxel size and half a voxel size root mean square (RMS) error [15] (contemporary CT cubic voxel size equals about one millimeter). However, revision cases often involve the presence of (failed) metal components, and image scatter caused by these metal parts can have a detrimental effect on the segmentation accuracy of the bone. With titanium alloys, the effect of metal artefacts on tissue density around a tightly fitting hip stem has been reported to gradually drop to zero at a range of 2 mm from the implant [30]. Unfortunately, to the author's knowledge, no similarly quantitative studies for the larger but fortunately thinner cup components in total hip arthroplasty are available.

Despite recent advances in imaging hardware and software filtering techniques [31, 32], the scatter effect in clinical CT can only be reduced, not neutralized. This finding applies to TrABL but also to other applications such as digital (2D or 3D) templating.

The use of TrABL for lower defect types (Paprosky II A-B-C), in which metal components are still in intimate contact with the host bone, requires caution.

Nevertheless, it should be noted that in revision surgery, especially the Paprosky types III A-B resulting from multiple revisions, the metal components have failed, that is, loosened and migrated, and are often even not in full nor in direct contact with the host bone. Regularly, the cup is moreover isolated from the latter by a layer of cement and/or bone graft. And if the cup is still in direct and stable contact, removal may implicate losing a thin contacting layer of bone. This counterweighs in a way the fact that the bone is segmented from outside the above-mentioned “drop range” around a metal implant. Furthermore, it should be stressed that the TrABL ratio is only influenced by *total* bone loss, and that a thin layer of bone will not provide initial stability to a hemispherical cementless acetabular component, in accordance to the basic question being posed in, for instance, the Paprosky defect classification [14].

Turning to *reconstruction accuracy* for pelvic defects, the algorithm used to set out and illustrate the generic TrABL methodology in this paper showed forth accuracy results of 3.2 ± 2.2 mm, 0.1 ± 1.0 mm, and 3.8 ± 2.9 degrees for the hip joint centrepoint, joint radius, and cup orientation, respectively, (mean ± standard deviation) [16]. Furthermore, in-depth analysis showed that these discrepancies can be attributed to the natural asymmetry which is present in the surface geometry of healthy contralateral bones [28].

Statistical shape models offer a valid alternative reconstruction method and can be looked after in the future. Just recently, this methodology has been applied to the hemipelvis with promising results [22]. Based on an initial database of twenty pelvic geometries and applied on a pelvis after tumour resection, the surface-matching quality proved submillimeter (mean 0.024 mm, max 0.115 mm).

## 4. Conclusions

A novel quantitative and computerized method was presented to assess the degree of total bone loss, measured in radial direction from the centre of the reconstructed acetabulum (abbreviated “TrABL”). Ingredients for this method are advanced 3D CT-based image processing and effective 3D anatomical reconstruction methodology which have previously been validated and published elsewhere in the literature. The method was implemented and applied on a first dataset of twelve hemi-pelves, mainly the Paprosky type IIIB. The quantitative output parameters are the TrABL ratio and graph, which can be used directly for comparison between specimens.

The novel method presented has the potential to facilitate the preoperative use of existing classifications and any directly related decision schemes that are used for clinical treatment selection. Precise and objective preoperative classification of bone defects will enable the clinician to make a proper selection from a complete range of implant solutions. 

An interesting topic for further research is to (either retro- or prospectively) look into the connection of the novel tool with, and the impact on, existing classification schemes and study results. Given the lower incidence of complex pelvic defects in the population, collaboration with other research groups is indicated to be able to generalize past and future study findings.

## Figures and Tables

**Table 1:** Total radial acetabular bone loss ratio for 12 specimens, (spec.: specimen, age (years), type: Paprosky et al.'s [4]. Subregions according to Figure 5. PostInf: posteroinferior, Inf: inferior, AntInf: anteroinferior, AntSup: anterosuperior, PostSup: posterosuperior, Med: medial. Min/Max: extremes of TrABL, for specimens (columns) and for regions (rows)).

Spec.	Age	Sex	Type	TrABL ratio [%]						Min	Max
				PostInf	Inf	AntInf	AntSup	PostSup	Med		
A	68	F	IIIb	0	8	58	22	28	0	0	58
B	50	F	IIIb	18	**55**	**83**	35	82	25	18	83
C	79	F	IIIb	28	18	41	18	29	34	18	41
D	66	M	IIIb	23	8	69	**53**	**92**	3	3	92
E	70	M	IIIb	29	17	56	22	50	19	17	56
F	66	M	IIIb	**42**	0	0	19	74	0	0	74
G	51	M	IIIb	14	2	0	12	61	0	0	61
H	60	F	IIIb	10	9	14	8	23	2	2	23
I	77	M	IIIb	27	24	7	3	66	**86**	3	86
J	68	F	IIIb	17	1	20	46	86	0	0	86
K	69	M	IIIb	14	9	10	17	62	17	9	62
L	59	F	IIc	10	2	0	5	25	12	0	*25*

			min	0	0	0	3	23	0		
			max	**42**	**55**	**83**	**53**	**92**	**86**		
